# Efficient mining of protein kinase structural data

**DOI:** 10.1186/1758-2946-5-S1-P16

**Published:** 2013-03-22

**Authors:** Stephen Maginn, Andrew Henry, Paul Labute, Johannes Maier, Nels Thorsteinson

**Affiliations:** 1 Chemical Computing Group, St Johns Innovation Centre, Cambridge CB4 0WS, UK; 2 Chemical Computing Group, 1010 Sherbrooke Street West, Montréal, Québec H3A 2R7, Canada

## 

Here, we introduce an aligned database of protein kinase structures that can be efficiently explored by sequence, structure, or by ATP pocket ligand (type or similarity). We also discuss an automated protocol for kinase identification, classification and superposition that relies on a curated reference set of structures and sequences covering the wide variety of human protein kinases.

